# Valorization of Mango By-Products: Bioactive Potential of Peel and Seeds and Their In Vitro Bioavailability

**DOI:** 10.3390/molecules31091462

**Published:** 2026-04-28

**Authors:** Sayonara Reyna, María de Guía Córdoba, María Ángeles Rivas, Iris Gudiño, María Vázquez-Hernández, Víctor Otero-Tuárez, Jaime Domínguez, Rocío Casquete

**Affiliations:** 1 Nutrición y Bromatología, Escuela de Ingenierías Agrarias, Universidad de Extremadura, Avd. Adolfo Suárez s/n, 06007 Badajoz, Spain; sayonara.reyna@gmail.com (S.R.); mrivasm@unex.es (M.Á.R.); igudino@unex.es (I.G.); mariavh@unex.es (M.V.-H.); rociocp@unex.es (R.C.); 2 Instituto Universitario de Investigación en Recursos Agrarios (INURA), Universidad de Extremadura, Avd. de la Investigación, 06006 Badajoz, Spain; 3 Facultad de Ciencias de la Vida y Tecnología, Universidad Laica Eloy Alfaro de Manabí, Ciudadela Universitaria, Manta 130802, Ecuador; victor.otero@uleam.edu.ec; 4 Secondary Education Institute IESO Los Barruecos, C. el Prado, 1, 10910 Malpartida de Cáceres, Spain; jdominguezm17@educarex.es

**Keywords:** mango by-products, phenolic compounds, antioxidant activity, antimicrobial activity, in vitro digestion

## Abstract

Mango (*Mangifera indica* L.) processing generates peel and seed by-products with high potential for valorization as sources of phenolic-rich ingredients. In this study, peel and seed from four Ecuadorian cultivars were extracted by ultrasound-assisted hydroalcoholic extraction and characterized for total phenolics, phenolic profile by HPLC-ESI-QTOF, antioxidant capacity (DPPH and ABTS), and antimicrobial activity against food-relevant bacteria. A dynamic in vitro gastrointestinal digestion model was also applied to evaluate digestion-driven changes in phenolic-related measurements and antioxidant response, and to assess colonic fermentation outputs, including short-chain fatty acids and viable microbial populations. The results showed a strong dependence on cultivar and by-product type, with total phenolics ranging from 2562.35 to 6304.35 mg GAE/100 g in peels and 212.69 to 3006.48 mg GAE/100 g in seeds. LC–MS profiles were dominated by gallotannin-related compounds and phenolic acids. Extracts displayed antioxidant activity (DPPH: 221.97–456.31 mg Trolox/100 g in peels; 43.71–530.46 mg Trolox/100 g in seeds) and dose-dependent antibacterial effects, with inhibition at 700 mg/L reaching 87.57–94.75%. Digestion markedly modulated phenolic-related indices and fermentation-associated metabolites, with peel phenolics decreasing from 284.27 to 73.95 mg GAE/L and seed extracts increasing propionic acid production up to 55.46 mM. Overall, mango peel and seed are differentiated, cultivar-sensitive sources of bioactive extracts with antioxidant and antimicrobial functionality and measurable impacts on colonic fermentation, supporting their use as sustainable ingredients for circular-economy food and nutraceutical applications.

## 1. Introduction

Mango (*Mangifera indica* L.) is a tropical fruit of great nutritional, economic, and industrial importance. It is primarily consumed as fresh pulp and is increasingly processed into juices, purees, dried products, and ingredients [1,2]. Globally, mango production (often reported alongside mangosteen and guava) has remained high, at approximately 62 million metric tons in the recent reference period and is projected to continue expanding during the current decade, with India as the leading producer [3,4]. In Ecuador, mango is a commercially important crop concentrated along the coastal strip, with well-documented production areas and reported technical development for provinces such as Manabí and Los Ríos [5,6].

Industrial processing generates a substantial amount of by-products, primarily peel and seeds, commonly estimated at approximately 35–60% of the whole fruit [7]. These by-products are frequently discarded despite their richness in recoverable dietary fiber, polyphenols, vitamins, carotenoids, lipids, and starches [8,9]. Given their composition, mango by-products have been increasingly investigated as sustainable sources of functional food and nutraceutical ingredients, as well as active/bio-based packaging additives, aligned with the goals of the circular economy [10,11,12,13].

Varietal effects are particularly relevant. Recent compositional studies have shown that peel fractions from different cultivars (Ataulfo, Panamanian, Manila, and Haden) differ in minerals, dietary fiber, pectin, and phenolic composition, with direct implications for functional standardization [14]. Furthermore, cultivar-specific differences have been linked to changes in the gastrointestinal behavior of mango phytochemicals, including their in vitro stability and bioaccessibility [15]. Therefore, controlling for cultivar origin and aligning processing strategies with target chemical classes are key steps for achieving reproducible antioxidant and antimicrobial effects [16].

Functionally, mango by-product extracts have been associated with dual antioxidant and antimicrobial potential. In food systems, mango peel powders or extracts have been used to mitigate lipid oxidation and improve the quality of meat products and bakery matrices, in line with their content of redox-active phytochemicals and matrix-stabilizing components [17,18]. In parallel, multiple studies report inhibitory effects against common foodborne pathogens and, in some cases, antibiofilm activity, reinforcing their relevance as natural antimicrobial sources [19,20,21].

Beyond the compositional profile and functionality of mango by-products, relevance depends on compound stability, bioaccessibility/bioavailability during digestion, and colonic bioconversion driven by the gut microbiota. Encapsulation and formulation strategies have been shown to improve the bioaccessibility of carotenoids and polyphenols from mango by-products, while processing technologies, such as high-pressure processing of mango juice, can further enhance phytochemical retention and gastrointestinal behaviour [15,22,23]. Under colonic conditions, dynamic fermentation models indicate the conversion of mango peel polyphenols into smaller phenolic acids (including hydroxybenzoic and phenylpropionic derivatives). Furthermore, bound phenolics associated with mango peel fiber may act as sustained-release reservoirs during digestion and fecal fermentation [24,25].

In this context, the objective of this study was to evaluate mango peel and seed extracts from four cultivars by combining compositional characterization with antioxidant and antimicrobial assays and by assessing their behaviour under simulated gastrointestinal digestion, monitoring changes in total phenolics and antioxidant activity across digestion stages and microbiota-associated outputs, including short-chain fatty acid production.

## 2. Results and Discussion

### 2.1. Phenolic Compounds of Extracts Obtained from the Peels and Seeds of Different Mango Varieties

Table 1 presents the total phenolic compounds (mg GAE/100 g dry sample) extracted from different mango varieties.

The mean total phenolic content (TPC) in mango peel ranged from 2562.35 to 6304.35 mg GAE/100 g dry sample, with significant differences among varieties within the peel fraction (*p* ≤ 0.05) (Table 1). Mantequilla exhibited the highest peel TPC (6304.35 mg GAE/100 g), followed by Chico y Grande (5862.16 mg GAE/100 g), whereas Manga showed the lowest value (2562.35 mg GAE/100 g). In seeds, TPC also differed significantly among varieties (*p* ≤ 0.05), ranging from 212.69 to 3006.48 mg GAE/100 g dry sample. Manga presented the highest seed TPC (3006.48 mg GAE/100 g), closely followed by Miguelillo (2861.64 mg GAE/100 g), while Mantequilla displayed markedly lower values (212.69 mg GAE/100 g).

Overall, these results highlight that TPC is strongly variety-dependent and differs between mango by-products, with peel and seed showing contrasting varietal rankings. This pattern is consistent with previous reports indicating that phenolic accumulation varies by genotype and tissue, and that different by-products may concentrate distinct phenolic pools [26]. For peel, Pacheco-Jiménez et al. [14] reported 2123–4851 mg GAE/100 g dry matter in Mexican cultivars, overlapping with the lower and intermediate peel values observed here and placing the highest peel means in our dataset above the upper end of that range. Kučuk et al. [8] reported 25.0 mg GAE/g dry weight in dried mango peel, consistent with the magnitude observed for the Manga peel fraction. Higher peel phenolic concentrations have also been described, supporting that peel can reach substantially elevated TPC depending on cultivar and sample set [27]. For seeds, Puscas et al. [28] reported 3893.33 mg GAE/100 g in mango seed powder, which is higher than the maximum observed in the present study but comparable in scale to the highest seed values found here. Differences across studies are expected due to cultivar, maturity stage, drying conditions, solvent system and extraction protocol.

The results of the identification and relative abundance of bioactive compounds in the mango extracts, derived from HPLC-ESI-QTOF analysis, are presented in Table 2 and Table 3. Table 2 systematically categorizes the identified constituents. Included for each entry are their respective retention times (Rt) and the characteristic fragments acquired during MSn mass spectrometry experiments. While Table 2 defines the presence of these compounds across all samples, their specific distribution and quantitative variations according to mango variety and by-product type are detailed in Table 3.

Table 2 shows that mango by-products are characterized primarily by phenolic acids and their derivatives, especially gallic acid (three isomers), galloyl glucose, hexagaloyl glucose, and ethyl gallate, along with a caffeic acid O-glucoside and a caffeoylquinic acid derivative. These compounds define a typical profile rich in gallotannins, which is consistent with findings in previousLC-MS studies [36,37] and highlighted in recent reviews [11,38]. In addition, several low-molecular-weight organic acids (succinic, citric, and multiple quinic acid isomers) are detected, along with a flavonoid (7-O-methyleriodictyol) and an unknown compound. As shown in Table 2, the chemical profile is dominated by hydrolyzable tannins and simple phenolic acids, with only one flavonoid annotated under the tested conditions. This composition aligns with recent descriptions of mango by-products as matrices rich in phenolic acids. These compounds offer significant technological potential, specifically as natural additives for stabilizing lipid-rich systems or as functional ingredients with antioxidant and antimicrobial properties in food and pharmaceutical formulations.

Table 3 summarizes the relative abundances (arbitrary area units) of the compounds listed in Table 2, organized by the main effects of the study factors: Extract (Factor E) and by-product (Factor B). While no statistically significant differences were observed by variety or by-product (*p* > 0.05), some clear numerical trends were noted. For example, caffeic acid O-glucoside (compound **1**) reaches 2151.93 units in the Mantequilla cultivar as a main effect, a much higher concentration than in the other cultivars, and quinic acid (compound **10**) shows, on average, an abundance approximately 2.4 times greater in the seeds (723.70) than in the peels (297.12). Regarding the by-product factor, the flavonoid 7-O-methyleriodictyol (compound **14**) is numerically higher in the peel (75.87) than in the seed (11.62), suggesting a tendency for higher relative abundances of several phenolic acids/galloylated compounds in seeds (compounds **1**, **3**–**5**, **7**, **8**, **10** and **11**), whereas the annotated flavonoid (compound **14**) was numerically higher in peel. A distribution that agrees with other observations on the composition of the peel versus the seeds in mango by-products [39,40].

Along the same lines, García et al. [26] analyzed mango agro-industrial waste and highlighted that gallic acid derivatives and other phenolic acids were among the main contributors to the functional value of these matrices, which agrees with our results, identifying gallic acid, galloyl glucose, and hexagaloyl glucose as predominant compounds. In another study, Hayat et al. [41] focused on mango seeds and demonstrated that gallic acid-rich extracts could be efficiently obtained by ultrasound-assisted extraction, highlighting the importance of the same class of compounds that appear as main peaks in our data. Similarly, Ojeda et al. [42] and Riaz et al. [43] applied deep eutectic solvents and ultrasound-assisted extraction, respectively, to mango by-products and reported high contents of phenolic acids and galloylated compounds, which is consistent with the compositional pattern observed in our Table 2 and Table 3.

### 2.2. Antioxidant Activity of Extracts

Antioxidant capacity was assessed using the 2,2-diphenyl-1-picrylhydrazyl (DPPH) and 2,2′-azinobis-(3-ethylbenzothiazoline)-6-sulfonic acid (ABTS) radical scavenging assays. The results are expressed as mg Trolox per 100 g of extract and are summarized in Table 4.

The mean antioxidant activity measured by DPPH in mango peel extracts ranged from 221.97 to 456.31 mg Trolox/100 g, with significant differences among cultivars. Chico y Grande (456.31 mg Trolox/100 g) and Manga (405.55 mg Trolox/100 g) showed the highest DPPH values in peel extracts, whereas Mantequilla presented the lowest (221.97 mg Trolox/100 g). In seeds, DPPH values ranged from 43.71 to 530.46 mg Trolox/100 g, also differing significantly among cultivars; Miguelillo exhibited the highest activity (530.46 mg Trolox/100 g), while Mantequilla showed a markedly low value (43.71 mg Trolox/100 g), indicating that cultivar ranking is not preserved between by-products. For ABTS, mean activities were more homogeneous: peel ranged from 47.80 to 62.07 mg Trolox/100 g (highest in Manga, 62.07 mg Trolox/100 g), and seeds ranged from 49.15 to 81.28 mg Trolox/100 g (highest in Mantequilla, 81.28 mg Trolox/100 g). No statistically significant differences were detected for ABTS among cultivars or between peel and seed in this dataset (Table 4).

As reference points in mango matrices, peel antioxidant activity in the same general magnitude has been reported previously; for instance, Plaitho et al. [44] cited DPPH Trolox activities for Sri Lankan mango peels of 11.86–18.91 µmol TE/g (297–474 mg Trolox/100 g), overlapping the upper part of the peel range observed here. In seed kernel–derived extracts, reported DPPH activities can be substantially higher depending on the extraction/fraction, with the lowest reported value of 54 µmol TE/g extract (1352 mg Trolox/100 g extract) in a supercritical-fluid-based study [45]. In the present study, the relationship between antioxidant activity and TPC was not uniform across all cultivars and by-products. Although higher TPC was associated with higher antioxidant activity in some cases, this pattern was not consistent, suggesting that antioxidant capacity was influenced not only by the total amount of phenolics but also by the qualitative composition of the extracts From a compositional standpoint, the antioxidant activity can be mainly attributed to the predominance of galloylated phenolics detected by HPLC-ESI-QTOF (Table 2 and Table 3), particularly gallic acid derivatives/isomers (compounds **3**–**6**), galloyl glucose (**2**), hexagalloyl glucose (**7**), and ethyl gallate (**8**), with an additional contribution from caffeic acid O-glucoside (**1**). Importantly, antioxidant activity has been demonstrated at the compound level for key constituents of this profile: gallic acid and ethyl gallate have been experimentally evaluated and shown to act as effective antioxidants [46]. In mango seed kernels specifically, the antioxidant response has been linked to the presence and relative abundance of individual phenolics, supporting galloylated constituents as major drivers of functional performance [45], which is consistent with the dominance of these compounds in the present profiles [7,47].

### 2.3. Antimicrobial Activity of Extracts

Antimicrobial activity was evaluated against a panel of food-relevant bacteria, including Listeria innocua, Listeria monocytogenes, Bacillus cereus, Staphylococcus aureus, Salmonella choleraesuis, and Escherichia coli. The antibacterial effects of mango by-products were assessed at concentrations of 700, 350, and 175 mg/L. The results, expressed as percentage inhibition, are summarized in Table 5, which is organized to show the main effects of the three factors studied: mango variety (Extract, E), by-product type (B), and concentration (C). Each value represents the mean performance of a factor level pro-averaged across all other experimental conditions, allowing for a clear statistical comparison of their individual influence on bacterial growth.

The antibacterial performance of mango by-product extracts (Table 5) showed a strain-dependent pattern, with overall inhibition values spanning 67.69–80.82% for *L. innocua*, 57.07–97.53% for *L. monocytogenes*, 10.38–94.54% for *B. cereus*, 67.55–81.83% for *S. aureus*, 67.73–80.22% for *S. choleraesuis*, and 65.43–75.90% for *E. coli*. The cultivar main effect was significant for *L. monocytogenes* and *B. cereus*. For *L. monocytogenes*, Manga reached the highest mean inhibition (97.53%), whereas Mantequilla showed the lowest (57.07%). For *B. cereus*, Chico y grande presented the highest inhibition (94.54%), while Mantequilla was markedly lower (10.38%), indicating that varietal performance can differ sharply depending on the target organism.

Regarding the main effect of the by-product, no significant by-product effect (Pb) was detected for any strain, although numerical differences between peel and seed were observed. Seeds tended to show higher mean inhibition for *L. innocua* and *S. aureus* (75.34% and 75.05%, respectively) than peels (66.15% and 68.45%), whereas peels showed higher mean inhibition for *L. monocytogenes* and *B. cereus* (87.93% and 83.55%) than seeds (78.85% and 66.69%). For *E. coli*, seed extracts also showed numerically higher inhibition than peel extracts (75.17% and 64.32%, respectively), although the by-product main effect was not significant. Importantly, the cultivar × by-product interaction (E × B) was significant for five of the six bacteria, but not for *E. coli*, indicating that differences between peel and seed were cultivar-specific rather than consistent in one direction across cultivars.

A pronounced dose–response was observed across all bacteria, with concentration showing a significant main effect in every case. Mean inhibition was highest at 700 mg/L (87.57–94.75% across strains), intermediate at 350 mg/L (71.24–85.70%), and lowest at 175 mg/L (45.49–65.18%). Interaction terms involving concentration were more limited: Pe × C was significant for *B. cereus* and *E. coli*, and was close to significance for *S. choleraesuis*.

The results obtained are consistent with previous research reporting the antibacterial activity of mango peel extracts against foodborne pathogens, including *S. aureus* and *B. cereus* [19,21]. From a compositional perspective (Table 2 and Table 3), the observed inhibition is consistent with the presence of galloylated phenolic fraction present in these extracts, particularly gallic acid isomers and higher gallotannins such as hexagaloyl glucose, along with ethyl gallate and caffeic acid-derived phenolics whose antimicrobial relevance has been demonstrated. Ethyl gallate has been experimentally shown to inhibit a panel of bacteria, with reported MIC and MBC values in the sub-mg/mL range, supporting its role as an antibacterial constituent when present in phenolic extracts [48]. Similarly, gallic acid has been reported to exert antibacterial and antibiofilm effects against *Staphylococcus aureus*, reinforcing the antimicrobial relevance of gallic acid-related structures such as those detected in these extracts [49]. In particular, purified gallotannins from mango seed (including multiple O-gallylglucose species) have been shown to exhibit antibacterial activity against food-relevant bacteria, supporting the role of higher galloylation structures as key contributors [50]. However, since no formal correlation analysis between individual compounds and antimicrobial activity was performed in the present study, a direct structure–activity relationship cannot be established. Taken together, these examples from the literature support a plausible compositional contribution of multiple galloylated/phenolic constituents to the inhibition values observed in Table 5, rather than demonstrating a single dominant causal compound.

### 2.4. Multivariate Analysis

**Figure 1:**
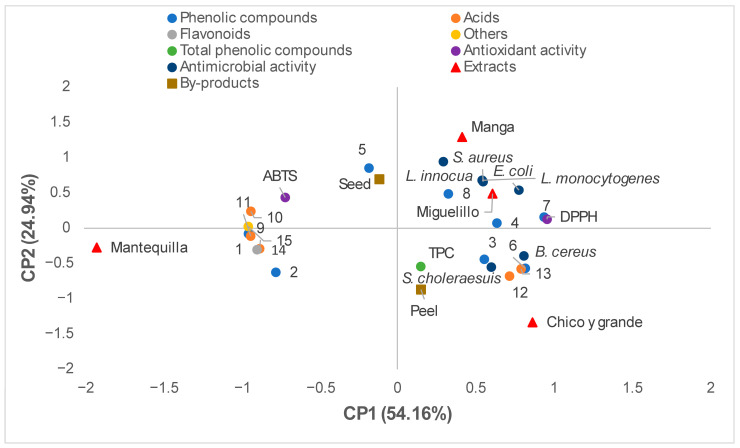
Principal Component Analysis (PCA) projection on the plane defined by PC1 (54.16%) and PC2 (24.94%). The displays the combined distribution of the analyzed variables and samples. The variables include phenolic compounds (**1**–**8**), organic acids (**9**–**13**), flavonoids (**14**), others (**15**), total phenolic compounds (TPC), antioxidant activity (DPPH and ABTS), and antimicrobial activity against *L. innocua*, *L. monocytogenes*, *B. cereus*, *S. aureus*, *S. choleraesuis*, and *E. coli*. The distribution of the samples according to mango varieties (Mantequilla, Manga, Miguelillo, and Chico y grande) and by-products (Peel and Seed). The numerical code for compounds corresponds to those identified in Table 2. This dispersion confirms that the mango extracts possess clearly differentiated phytochemical profiles and biological capacities, where the peel and the varieties on the right side of the plot stand out for their antimicrobial and antioxidant potential.

Principal Component Analysis (PCA) integrates the distribution of variables and samples into a plane defined by PC1 (54.16%) and PC2 (24.94%), which together explain 79.10% of the total variance (Figure 1). PC1 differentiates the samples primarily based on their antimicrobial capacity and phenolic content. Variables related to antimicrobial activity against pathogens such as *S. aureus*, *L. monocytogenes*, *L. innocua*, and *E. coli*, as well as antioxidant activity measured by DPPH, project toward the right side of the plot (positive PC1 values). The Manga, Miguelillo, and Chico y grande varieties are positioned in this region, suggesting higher biological efficacy and phytochemical richness. In contrast, the Mantequilla variety is located on the opposite side (left), associating with compounds such as caffeic acid O-glucoside (peak **1**) and the flavonoid 7-O-methyleriodictyol (peak **14**), but showing a lower correlation with the aforementioned biological activities.

On the other hand, PC2 separates the samples according to their by-product profile and acid types. The Seed by-product is situated in the upper quadrant, showing a relationship with a caffeoylquinic acid derivative (peak **5**) and ABTS antioxidant activity. In contrast, the Peel by-product and the Chico y grande variety shift toward the lower quadrants, associating more closely with gallic acid (peak **6**), quinic acids (peaks **12** and **13**), and activity against *B. cereus* and *S. choleraesuis*.

### 2.5. Characterisation of Digestion Extracts

Table 6 summarizes the changes in total phenolic content (TPC) and antioxidant capacity (ABTS and DPPH) of mango by-product extracts during an in vitro simulated gastrointestinal digestion. Measurements were taken for the undigested extract and after the gastric, small-intestinal, and colonic phases. For the colonic step, data are reported as a single pooled value because no significant differences were detected among its sub-stages for either TPC or antioxidant activity.

Table 6 shows a clear phase-dependent evolution of TPC and antioxidant capacity during simulated gastrointestinal digestion, with distinct behaviors for the peel and seed extracts. TPC was highest in the peel during the initial and gastric stages, remaining virtually unchanged from 284.27 to 284.25 mg GAE/L, before decreasing to 146.99 mg GAE/L in the small intestine phase and to 73.95 mg GAE/L in the colon simulation. The seed extract showed an initial TPC of 96.13 mg GAE/L, decreased after gastric digestion to 60.58 mg GAE/L, remained similar in the small intestine at 62.51 mg GAE/L, and decreased to 31.71 mg GAE/L in the colon phase. The control remained low during the small intestine phase (27.54, 21.55, and 17.49 mg GAE/L) but increased markedly in the colon simulation to 63.21 mg GAE/L. Differences between samples were significant for TPC at each stage of digestion.

ABTS values remained low for the peel and seed extracts throughout digestion, while the control showed consistently higher values, with significant differences between samples at all stages. DPPH activity was detectable in the initial sample and after gastric simulation, especially for the peel extract, but decreased to undetectable levels in the small intestine and colon phases for all samples.

Overall, the marked decrease in TPC following intestinal simulation and the strong phase dependence of antioxidant indices are consistent with previous in vitro digestion studies in mango matrices, which report substantial changes in phenolic-related measurements throughout the gastrointestinal stages and changes in the colonic phase that can alter the pool of Folin-reactive components [51,52]. This framework is compatible with phenolic profiles rich in high-molecular-weight polyphenols (hydrolyzable tannins), whose bioaccessibility and measured responses can be strongly influenced by the digestive environment and matrix effects [53]. Comparable phase-dependent changes in antioxidant capacity have also been described for mango-derived products subjected to in vitro digestion [54].

Table 7 reports short-chain fatty acid (SCFA) formation during the simulated colonic stage.

Table 7 shows that the phenolic extracts of mango peel and seed significantly modify the acetic and propionic acid profiles throughout the simulated colon compared to the control. Regarding acetic acid, the highest concentrations in the proximal colon were observed with the seed extract (1.42 mM), followed by the peel (1.14 mM) and the control (0.36 mM), while in the distal region the peel reached the highest value (11.65 mM) and the seed the lowest (8.87 mM). These results are consistent with in vitro gastrointestinal digestion and colonic fermentation studies in which mango ingredients rich in dietary fiber and phenolic compounds (mango-based fruit bars, predigested peel, or peel pectin) induce marked acetic acid formation and an increase in total SCFAs [51,55,56]. Similarly, probiotic yogurt enriched with mango peel powder shows intense biotransformation of phenolic compounds, accompanied by a marked increase in SCFAs after in vitro digestion and colonic fermentation, reinforcing the direct role of phenolic extracts from mango peel in SCFA production [57].

In the case of propionic acid, Table 7 shows a more selective response: the phenolic extracts of mango seed produces significantly higher concentrations in all three colonic regions (33.75 mM, 55.46 mM and 55.31 mM) than the peel extract and the control, among which no significant differences were observed. This indicates that the phenolic profile of the seed specifically stimulates propionate-producing microbial pathways, consistent with the strong dependence of SCFA patterns on the structure of dietary fiber and bound polyphenols, as described for mango by-products [25,26]. Comparable shifts toward propionate-enriched profiles or high SCFA release have also been described in other agro-industrial by-products rich in phenolic compounds. Reyna et al. [58] used combined peel and seed extracts from papaya by-products, which significantly improved acetate and propionate generation during simulated colonic fermentation. From a physiological perspective, the differential contribution of these phenolic extracts to the production of acetate or propionate is especially relevant, since both SCFAs exert distinct but synergistic functions in energy supply, lipid regulation and glucose homeostasis, acting as substrates and signaling molecules in metabolically active tissues [59,60].

Table 8 summarizes the evolution of the microbiology for mango peel and seed extracts during simulated in vitro gastrointestinal digestion, presenting the values for the initial sample and for the small intestine, and colonic phases. For the colonic stage, the results are presented as a single pooled value because no significant differences were detected between its substages.

Table 8 shows that microbial counts were modulated by the extracts in a stage-dependent manner. In the small-intestine simulation, total viable bacteria were lower with the peel extract (7.41 log CFU/mL) than in the control (8.21) and seed (8.03), and enterobacteria followed the same pattern (control 8.23; peel 7.35; seed 7.73). In the proximal colon, total viable bacteria were highest in the control (8.76) and lower in peel (8.23) and seed (8.12), whereas in the distal colon the peel treatment showed the highest mean for total viable bacteria (9.04). Lactic acid bacteria and enterococci were not detected in the small-intestine stage, but were detected across the colon stages; for both groups, the control generally showed higher means than peel and seed, with the lowest counts frequently observed in the seed treatment, particularly in the distal colon for lactic acid bacteria (control 6.37; peel 5.34; seed 4.54) and enterococci (control 6.30; peel 5.44; seed 4.48).

The reductions observed for enterobacteria and, in several colonic stages, for lactic acid bacteria/enterococci are consistent with the selective pressure that dietary polyphenols and tannin-rich fractions can exert on intestinal bacteria, with effects that depend on concentration, matrix, and fermentation stage [61]. This interpretation aligns with evidence that tannin-type compounds can inhibit or delay growth in Lactobacillus under relevant in vitro conditions, supporting the plausibility of lower lactic acid bacteria counts in polyphenol-exposed systems [62]. In mango-specific settings, predigested mango peel has been shown to modulate the gut microbiota in a dynamic colon model (TIM-2), indicating that mango-derived constituents can influence community structure during colonic fermentation [55]. In addition, the galloylated/gallic-acid–related pool typical of mango by-products is also compatible with microbial transformation capacity, since a broad range of human gut bacteria can metabolize gallic acid, implying that community responses may shift over time as substrates are converted [63]. Finally, in vivo data support that mango intake can modulate gut microbial ecology and fermentation outputs, reinforcing the biological plausibility of stage-dependent microbial changes observed in vitro [64]. However, these changes should not be interpreted as inherently beneficial. In particular, the reduction in lactic acid bacteria may reflect either selective microbial modulation or a potentially undesirable antimicrobial effect, depending on the microbial target and physiological context [61,62]. Therefore, the biological significance of these changes and their implications for safe use should be interpreted cautiously.

## 3. Materials and Methods

### 3.1. Plant Material

Mango by-products (peels and seeds) from four commercial cultivars (Chico y grande, Miguelillo, Manga, and Mantequilla) were collected at commercial maturity from smallholder orchards in Ecuador. After harvesting, the peels and seeds were separated from the fruits, thoroughly washed to remove any adhering pulp, and freeze-dried (LyoBeta, Telstar, Barcelona, Spain). The freeze-drying process consisted of a freezing step at −40 °C for 4 h, followed by primary and secondary drying (8.5 h at −20 °C and 6.5 h at 20 °C, respectively) at 400 µbar. The dried material was ground into a fine powder and stored in airtight containers at room temperature until use.

### 3.2. Bacterial Strains

Six reference bacterial strains obtained from the Spanish Type Culture Collection (CECT) were used in antimicrobial assays. These included *Staphylococcus aureus* CECT 976, *Bacillus cereus* CECT 131, *Listeria monocytogenes* CECT 911, *Listeria innocua* CECT 910, *Salmonella choleraesuis* CECT 4395, and *Escherichia coli CECT 4267*.

### 3.3. Extraction and Determination of Total Phenolic Content

Phenolic compounds were recovered by ultrasound-assisted extraction (UAE) with 80% (*v*/*v*) ethanol in water. Briefly, 10 g of dried peel or seed powder were mixed with 60 mL of solvent (solid:liquid ratio = 1:6 *w*/*v*) and sonicated in a water bath (40 kHz, 45 °C) for 60 min. The suspension was filtered (Whatman No. 1, Buckinghamshire, UK), and the retained solids were re-extracted once with an additional 60 mL under identical conditions. The combined filtrates were concentrated under reduced pressure to remove ethanol and topped up with distilled water (aqueous extract). The extracts were aliquoted and kept at −20 °C.

Total phenolic content (TPC) was determined by the Folin–Ciocalteu colorimetric assay. The procedure followed Casquete et al. [65]: aliquots of each extract were combined with Folin–Ciocalteu reagent, saturated Na_2_CO_3_ solution, and Milli-Q water. After incubation for 60 min at room temperature in the dark, absorbance was recorded at 760 nm. The results were expressed as milligrams of gallic acid equivalents (GAE) per 100 g of dry weight of the original peel or seed material.

### 3.4. LC–MS Identification of Bioactive Compounds

Compound profiling was carried out by HPLC–QTOF–MS. Extracts were diluted with LC–MS–grade methanol to 100 µg mL^−1^ and clarified through 0.45 µm syringe filters, then introduced into an HPLC system coupled to a quadrupole time-of-flight mass spectrometer (Agilent 6530 QTOF; Agilent Technologies, Palo Alto, CA, USA). Separation was performed on a C18 column (4.6 × 150 mm, 4.8 µm). Detection and initial identification of the bioactive compounds were performed with a quadrupole time-of-flight (Q-TOF) tandem mass analyzer, utilizing an electrospray ionization (ESI) source in negative mode. Provisional compound identities were assigned through consultation with the MassBank database [29].

### 3.5. Antioxidant Capacity

Antioxidant activity was quantified using two spectrophotometric assays. Extracts were evaluated for their capacity to decolorize the 1,1-diphenyl-2-picrylhydrazyl (DPPH) radical, following Teixeira et al. [66], and to quench the 2,2′-azinobis (3-ethylbenzothiazoline-6-sulfonic acid) radical cation (ABTS), according to Re et al. [67]. Results were reported as milligrams of Trolox equivalents per 100 g of extract.

### 3.6. Antimicrobial Activity

The antimicrobial effects of the mango extracts were assessed by a microplate broth microdilution assay with kinetic monitoring of bacterial growth [68]. Each bacterial strain was grown in Brain Heart Infusion (BHI) broth at 37 °C for 24 h prior to the assay. The inoculum for each test was standardized to 10^5^ CFU/mL in fresh BHI. In sterile 96-well microplates, 50 µL of the standardized inoculum were combined with 50 µL of serial dilutions of each mango extract in BHI. Solvent controls (BHI with an equivalent final concentration of ethanol ≤ 1% *v*/*v*) and growth controls (BHI with inoculum but no extract) were included in each assay. The microplates were incubated at 37 °C in a plate reader that recorded OD_600_ every hour for 24 h. Growth curves were obtained for each condition, and growth inhibition at each time point was calculated relative to the uninhibited control.

### 3.7. Simulated Gastrointestinal Digestion

A dynamic in vitro gastrointestinal digestion system was used to simulate the fate of extracts in the gastric, intestinal, and colonic phases, adapted from the model described by Rivas et al. [69]. The system consisted of three jacketed and stirred bioreactors (BIOSTAT^®^ A, Sartorius, Göttingen, Germany) connected in series to sequentially simulate the stomach, small intestine, and colon. The reactors were maintained at 37 °C with continuous stirring (150 rpm) under low oxygen conditions (<0.5% O_2_). The pH in the gastric compartment was maintained at 2.5, adjusted to 6.5 in the small intestine compartment, and gradually increased from 5.7 to 6.8 in the colonic compartment to replicate conditions throughout the colon. Each digestion experiment (test system) used 250 mL of mango peel or seed extract solution from the Manga variety (at 750 ppm in 1% gelatin in PBS) introduced into the gastric reactor. This cultivar was selected for the digestion study because it showed one of the most relevant bioactive profiles among the four cultivars evaluated, including the highest total phenolic content in the seed fraction and high antioxidant and antimicrobial activities. A parallel control experiment was performed using 250 mL of the 1% gelatin/PBS matrix without added extract. Before initiating digestion, the colon reactor was inoculated with 25 mL of Man, Rogosa, Sharpe (MRS) broth and 10 mL of human fecal inoculum to establish the colonic microbiota. This colonic phase was allowed to stabilize for 12 h before introducing the intestinal effluent. A simulated digestion was initiated by adding pepsin to the gastric vessel to reach a final concentration of 4.5% (*w*/*v*). As the gastric contents emptied into the intestinal reactor, pancreatin 3% (*w*/*v*) and bile salts 7.5% (*w*/*v*) were automatically introduced into the intestinal vessel. Subsequently, the effluent from the intestinal phase was fed to the colonic reactor, where the resident microbiota fermented. Each complete digestion was performed in duplicate for each extract type (peel and seed).

Samples were collected from the reactors at key stages: initially (before enzyme addition, representing the undigested extract in the matrix), after the gastric phase, after the intestinal phase, and during the colonic phase at 8 h, 20 h, and 36 h (corresponding to the ascending, transverse, and descending colon conditions, respectively). These samples were immediately processed as described below. It should be noted that the analyte concentrations measured in each reactor were not corrected for the progressive dilutions inherent in the dynamic system (approximately 5.8-fold dilution upon reaching the stomach, 8.3-fold upon reaching the small intestine, and 10.9-fold upon reaching the colon).

### 3.8. Characterization of Digestion Samples

Upon collection, digestion samples were handled according to their intended analyses. Liquid samples for chemical analyses were centrifuged at 10,000× *g* for 10 min at 4 °C to remove particulate matter and then filtered through 0.45 µm nylon membranes. Samples for microbiological analysis were not centrifuged or filtered to preserve the live bacteria.

The following analyses were performed on the digestion samples:

Total phenolic content (TPC): Measured using the Folin–Ciocalteu assay as described in Section 3.3, to determine the concentration of phenolic compounds at each digestion stage.
Antioxidant capacity: Evaluated by the DPPH and ABTS radical scavenging assays (Section 3.5) to monitor changes in antioxidant activity through digestion.
Short-chain fatty acids (SCFAs): Quantified by gas chromatography with flame ionization detection (GC-FID, Shimadzu GC-2010 Plus, Kyoto, Japan) following the method of Rivas et al. [70]. Prior to injection, samples were mixed with 2-ethylbutyric acid as an internal standard. SCFA concentrations were calculated from the ratio of each analyte’s peak area to that of the internal standard, using calibration curves for individual acids.
Viable microbiota counts: Enumerated by plating serial dilutions of each sample on selective and non-selective agar media. Specifically, aliquots (0.1 mL) of decimal dilutions in sterile 1% peptone water were spread onto Plate Count Agar (PCA) for total aerobic mesophilic bacteria (incubated at 30 °C for 48 h), MRS agar (pH 5.6) for lactic acid bacteria (30 °C, 48 h), Slanetz & Bartley agar for enterococci (37 °C, 48 h), and Violet Red Bile Glucose (VRBG) agar for enterobacteria (30 °C, 24 h). After incubation, colonies were counted and results were expressed as logarithmic colony-forming units per mL of sample (log CFU/mL).

### 3.9. Statistical Analysis

All data were analyzed using SPSS Statistics v21.0 (IBM Corp., Armonk, NY, USA). Descriptive statistics (mean ± standard deviation) were calculated for all measured parameters. Group differences (e.g., between mango cultivars, between peel vs. seed extracts, or other factors) were evaluated by one-way, two-way, or three-way analysis of variance (ANOVA) as appropriate. When ANOVA indicated significant effects (*p* ≤ 0.05), post hoc pairwise comparisons were performed using Tukey’s Honest Significant Difference (HSD) test. Differences in time-course measurements between extract-treated samples and controls over the digestion period were assessed by repeated-measures ANOVA, and Bonferroni correction was applied for multiple comparisons (*p* ≤ 0.05 considered significant). Finally, to explore multivariate patterns in the dataset, a principal component analysis (PCA) was conducted on the correlation matrix of the standardized variables, allowing visualization of relationships and clustering among the different treatment conditions and response variables.

## 4. Conclusions

In conclusion, mango by-products exhibit a composition and functionality strictly dependent on the cultivar and the matrix type (peel or seed). While the peel of the Chico y grande, Manga, and Miguelillo varieties stands out for its phenolic richness and antimicrobial efficacy, the seed displays specific phytochemical profiles (such as caffeoylquinic acid derivatives) associated with high antioxidant capacity. These differences result in dose-dependent bacterial inhibition that varies according to the specific cultivar-by-product combination. During digestion and colonic fermentation, the extracts undergo significant transformations: the peel favors acetate production, while the seed promotes propionate production. Overall, these results validate the valorization of these residues as sustainable sources of natural ingredients, suggesting that the strategic selection of variety and by-product allows for the optimization of their application in food and nutraceutical contexts according to the desired bioactive profile. Nevertheless, before considering their incorporation into food or nutraceutical applications, further studies are needed to assess their safety, including toxicity, tolerance, and dose-dependent effects under relevant conditions.

## Figures and Tables

**Table 1:** Total phenolic compounds (mg GAE/100 g dry sample) of the extracts obtained from different mango varieties.

Extract	Peel			Seed			
	Mean		SD *	Mean		SD	*p*-Values
Chico y grande	5862.16 ^c^	±	74.78	933.63 ^b^	±	34.23	<0.001
Miguelillo	5162.41 ^b^	±	302.52	2861.64 ^c^	±	70.82	0.004
Manga	2562.35 ^a^	±	94.93	3006.48 ^c^	±	136.77	0.013
Mantequilla	6304.35 ^c^	±	173.95	212.69 ^a^	±	23.40	<0.001
*p*-Values	<0.001			<0.001			

* SD: standard deviation. Superscripts (a–c): among varieties within the same by-product (*p* ≤ 0.05).

**Table 2:** Identification of the main compounds from mango by-products extracts analyzed by HPLC-ESI-QTOF.

Peak	Rt (min)	[M-H]^−^	MS/MS (*m*/*z*)	Compounds Identified
Phenolic Compounds				
1 *	4.465	341	179, 135, 101	Caffeic acid O-glucoside ^1,2^
2 *	7.316	331	125, 169	Galloyl glucose ^1,2,3^
3 *	15.63	169	125	Gallic acid (isomer 1) ^1,2,3^
4 *	15.81	169	125	Gallic acid (isomer 2) ^1,2,3^
5 ****	15.88	677.5	250, 132	caffeoylquinicacid derivated ^4^
6 *	15.94	169	125	Gallic acid (isomer 3) ^1,2,3^
7 ****	15.96	1091	939	Hexagalloyl glucose ^5^
8 *	16.95	197	169, 125	Ethyl gallate ^1,2,3^
Acids				
9 ***	4.500	117	–	Succinic Acid ^6^
10 *	4.564	191	127, 173	Quinic acid (Isomer 1) ^1,2,7^
11 *	6.355	191	127, 111	Citric acid ^1,7^
12 *	10.02	191	127, 173	Quinic acid (Isomer 1) ^1,2,7^
13 *	14.31	191	127, 173	Quinic acid (Isomer 1) ^1,2,7^
Flavonoids				
14 **	14.65	301	135	7-O-Methyleriodictyo ^2^
Others				
15	4.168	128	114	Unknown

^1^ MassBank [29]; ^2^ Tan et al. [30]; ^3^ Dorta et al. [31]; ^4^ Kramberger et al. [32]; ^5^ Sandhu [33]; ^6^ Serrano-García et al. [34]; ^7^ Zhao et al. [35]. * Reported spectrum in mango in bibliography and corroborated in massbank. ** Reported spectrum in bibliography. *** Reported compound in bibliography and corroborated in massbank. **** Reported spectrum in other foods in bibliography.

**Table 3:** Relative abundances in arbitrary area units of the main compounds from mango by-products extracts analyzed by HPLC-ESI-QTOF.

Factor	Extracts (E)				By-Products (B)		*p*-Values	
Levels ^1^	Chico y Grande	Manga	Mantequilla	Miguelillo	Peel	Seed	Pe	Pb
Phenolic Compounds								
1	313.85	101.15	2151.93	422.02	419.40	1075.08	0.361	0.490
2	8.13	-	16.82	1.12	8.54	4.49	0.129	0.540
3	59.21	27.77	31.41	51.16	37.76	47.01	0.518	0.597
4	88.53	61.63	48.94	91.34	55.48	89.74	0.760	0.251
5	51.18	70.07	67.10	76.61	60.26	72.22	0.673	0.401
6	132.62	101.09	80.35	108.44	117.49	93.75	0.960	0.701
7	53.68	48.13	21.14	49.12	37.93	48.10	0.821	0.685
8	34.21	33.99	25.17	81.36	20.36	67.00	0.510	0.067
Acids								
9	8.68	-	38.60	15.52	16.60	14.80	0.348	0.915
10	143.93	536.55	1207.09	154.07	297.12	723.70	0.252	0.348
11	14.43	9.51	40.89	12.21	13.48	25.04	0.280	0.397
12	87.83	16.09	-	37.48	48.17	22.53	0.413	0.522
13	17.10	4.24	-	9.22	9.24	6.04	0.383	0.675
Flavonoids								
14	7.86	14.89	147.57	4.65	75.87	11.62	0.460	0.387
Others								
15	1.58	1.45	120.11	5.74	5.25	59.20	0.436	0.381

^1^ The numerical code corresponds to the compounds listed in Table 2. Values represent the marginal means for each factor level. Factor E averages across both by-products, while Factor B averages across all four mango extracts.

**Table 4:** Antioxidant activity determined by DPPH and ABTS methods (mg Trolox/100 g) of extracts obtained from by-products of different mango varieties.

Extracts	DPPH							ABTS						
	Peel			Seed				Peel			Seed			
	Mean		SD ^1^	Mean		SD	*p*-Value	Mean		SD	Mean		SD	*p*-Value
Chico y grande	456.31 ^d,2^	±	30.63	267.34 ^b^	±	1.96	0.008	47.80	±	30.17	54.82	±	24.72	0.771
Miguelillo	322.30 ^b^	±	6.59	530.46 ^d^	±	39.10	0.010	51.04	±	13.95	72.92	±	13.17	0.120
Manga	405.55 ^c^	±	8.85	332.74 ^c^	±	8.99	<0.001	62.07	±	3.21	49.15	±	7.20	0.072
Mantequilla	221.97 ^a^	±	6.80	43.71 ^a^	±	2.32	<0.001	52.53	±	2.70	81.28	±	12.56	0.052
*p*-Values	<0.001			<0.001				0.760			0.110			

^1^ SD: standard deviation. ^2^ Superscripts (a–d): among varieties within the same by-product (*p* ≤ 0.05).

**Table 5:** Antibacterial activity against different bacteria (% inhibition) of the different extracts obtained from the by-products of different mango varieties.

	*L. innocua*			*L. monocytogenes*			*B. cereus*			*S. aureus*			*S. choleraesuis*			*E. coli*		
	Mean		SD ^1^	Mean		SD	Mean		SD	Mean		SD	Mean		SD	Mean		SD
Extract (E)																		
Chico y grande	67.69	±	17.36	82.17 ^b^	±	23.19	94.54 ^b^	±	10.93	71.17	±	17.91	77.27	±	13.90	65.43	±	20.99
Miguelillo	74.26	±	14.77	86.16 ^b^	±	22.47	76.14 ^b^	±	35.23	72.69	±	15.19	80.22	±	14.52	75.90	±	16.67
Manga	80.82	±	14.60	97.53 ^b^	±	3.89	82.11 ^b^	±	27.77	81.83	±	16.09	67.73	±	12.04	72.27	±	21.97
Mantequilla	68.95	±	16.57	57.07 ^a^	±	11.28	10.38 ^a^	±	16.33	67.55	±	16.55	79.80	±	11.77	74.39	±	32.53
By-products (B)																		
Peel	66.15	±	16.24	87.93	±	22.95	83.55	±	26.17	68.45	±	14.48	77.18	±	11.77	64.32	±	16.36
Seed	75.34	±	15.29	78.85	±	21.98	66.69	±	41.72	75.05	±	17.15	77.04	±	16.96	75.17	±	23.26
Concentration (C) (mg/L)																		
700	87.57 ^c^	±	6.40	94.75 ^b^	±	11.32	88.53 ^b^	±	26.86	91.02 ^b^	±	7.48	92.37 ^c^	±	9.53	93.63 ^c^	±	9.57
350	74.52 ^b^	±	8.16	85.70 ^b^	±	19.87	82.91 ^b^	±	38.75	71.70 ^b^	±	8.55	77.82 ^b^	±	6.95	71.24 ^b^	±	12.60
175	54.74 ^a^	±	10.51	65.18 ^a^	±	23.94	45.49 ^a^	±	33.38	55.83 ^a^	±	7.02	61.07 ^a^	±	8.11	49.79 ^a^	±	13.64
*p*-Values																		
Pe	0.383			0.007			<0.001			0.482			0.412			0.687		
Pb	0.104			0.257			0.210			0.261			0.978			0.158		
Pc	<0.001			<0.001			0.006			<0.001			<0.001			<0.001		
Pe * b	0.016			<0.001			<0.001			<0.001			0.028			0.958		
Pe * c	0.775			0.351			<0.001			0.570			0.053			0.001		
Pb * c	0.899			0.645			0.593			0.287			0.254			0.328		

^1^ SD: standard deviation of the main effects for each factor (Variety, By-product, and Concentration). Superscripts (a–c): indicate statistical differences (*p* ≤ 0.05) between levels within the same column and factors. The symbol * is used to indicate the interaction between two factors.

**Table 6:** Total phenolic compounds (mg GAE/L) and antioxidant capacity (ABTS and DPPH methods) (mg Trolox/L) of mango extracts during simulated in vitro gastrointestinal digestion of the “Manga” variety.

Parameter	Sample	Initial Sample			Stomach Simulation			Small Intestine Simulation			Colon Simulation		
		Mean		SD ^1^	Mean		SD	Mean		SD	Mean		SD
TPC ^2^	Control	27.54 ^a,3^	±	0.60	21.55 ^a^	±	0.35	17.49 ^a^	±	1.38	63.21 ^b^	±	2.40
	Peel	284.27 ^c^	±	6.00	284.25 ^c^	±	2.30	146.99 ^c^	±	16.02	73.95 ^b^	±	18.82
	Seed	96.13 ^b^	±	8.54	60.58 ^b^	±	2.38	62.51 ^b^	±	3.66	31.71 ^a^	±	2.20
	*p*-Values	<0.001			<0.001			<0.001			0.008		
ABTS	Control	0.43 ^b^	±	0.02	0.45 ^b^	±	0.04	0.91 ^b^	±	0.03	0.61 ^c^	±	0.01
	Peel	0.06 ^a^	±	0.03	0.05 ^a^	±	0.01	0.11 ^a^	±	0.07	0.11 ^b^	±	0.01
	Seed	0.03 ^a^	±	0.02	0.09 ^a^	±	0.06	0.03 ^a^	±	0.02	0.07 ^a^	±	0.02
	*p*-values	<0.001			<0.001			<0.001			<0.001		
DPPH	Control	0.17 ^a^	±	0.01	0.15 ^b^	±	0.05	0.00	±	0.00	0.00	±	0.00
	Peel	0.99 ^b^	±	0.23	0.80 ^c^	±	0.041	0.00	±	0.00	0.00	±	0.00
	Seed	0.25 ^a^	±	0.12	0.00 ^a^	±	0.00	0.00	±	0.00	0.00	±	0.00
	*p*-Values	0.001			0.001			0.108			0.083		

^1^ SD: standard deviation; ^2^ TPC: total phenolic compounds; ^3^ different superscript letters (a–c) indicate significant differences between samples (*p* ≤ 0.05).

**Table 7:** SCFA (mM) production from mango extracts during simulated in vitro gastrointestinal digestion of the “Manga” variety.

SCFAs	Sample	Colon Simulation (Stage)								
		Proximal			Transverse			Distal		
		Mean		SD ^1^	Mean		SD	Mean		SD
Acetic acid	Control	0.36 ^a,2^	±	0.05	7.79 ^a^	±	0.32	10.34 ^b^	±	0.03
	Peel	1.14 ^b^	±	0.05	10.81 ^c^	±	0.06	11.65 ^c^	±	0.15
	Seed	1.42 ^c^	±	0.01	9.61 ^b^	±	0.16	8.87 ^a^	±	0.17
	*p*-Values	<0.001			<0.001			<0.001		
Propionic acid	Control	19.70 ^a^	±	0.12	43.41 ^a^	±	0.22	50.29 ^a^	±	1.13
	Peel	18.73 ^a^	±	0.51	42.77 ^a^	±	0.39	50.82 ^a^	±	0.75
	Seed	33.75 ^b^	±	0.16	55.46 ^b^	±	1.36	55.31 ^b^	±	1.02
	*p*-Values	<0.001			<0.001			0.001		

^1^ SD: standard deviation. ^2^ Different superscript letters (a–c) indicate significant differences between samples (*p* ≤ 0.05).

**Table 8:** Main microbial population counts (CFU/mL) from mango extracts during simulated in vitro gastrointestinal digestion of the “Manga” variety.

Microbial Population	Sample	Small Intestine Simulation			Colon Simulation (Stage)								
					Proximal			Transverse			Distal		
		Mean		SD ^1^	Mean		SD	Mean		SD	Mean		SD
Total viable bacteria	Control	8.21 ^b^	±	0.07	8.76 ^b^	±	0.09	8.34	±	0.01	8.31	±	0.08
	Peel	7.41 ^a^	±	0.15	8.23 ^a^	±	0.06	8.28	±	0.13	9.04	±	1.45
	Seed	8.03 ^b^	±	0.19	8.12 ^a^	±	0.02	7.69	±	0.29	7.48	±	0.22
	*p*-values	0.026			0.004			0.069			0.326		
Enterobacteria	Control	8.23 ^c^	±	0.02	8.72	±	0.14	8.33	±	0.08	8.39	±	0.07
	Peel	7.35 ^a^	±	0.11	8.51	±	0.32	8.17	±	0.07	7.95	±	0.40
	Seed	7.73 ^b^	±	0.01	7.98	±	0.05	8.07	±	0.12	7.54	±	0.15
	*p*-values	0.002			0.081			0.153			0.097		
Lactic acid bacteria	Control	0.00	±	0.00	6.38 ^c^	±	0.04	6.29 ^b^	±	0.01	6.37 ^c^	±	0.02
	Peel	0.00	±	0.00	5.86 ^b^	±	0.05	5.91 ^b^	±	0.24	5.34 ^b^	±	0.25
	Seed	0.00	±	0.00	5.65 ^a^	±	0.04	5.34 ^a^	±	0.26	4.54 ^a^	±	0.10
	*p*-values	-			0.001			0.043			0.003		
*Enterococci*	Control	0.00	±	0.00	6.51	±	0.03	6.59 ^c^	±	0.08	6.30 ^c^	±	0.18
	Peel	0.00	±	0.00	6.03	±	0.03	6.22 ^b^	±	0.09	5.44 ^b^	±	0.02
	Seed	0.00	±	0.00	6.71	±	0.28	5.33 ^a^	±	0.04	4.48 ^a^	±	0.16
	*p* values	-			0.056			0.001			0.002		

^1^ SD: standard deviation. Different superscript letters (a–c) indicate significant differences between samples (*p* ≤ 0.05).

## Data Availability

Data Availability Statements are available in sections of this paper.
